# Comparison study of LANJA, UE, and VDO video laryngoscopes for simulated endotracheal intubation in beginners

**DOI:** 10.1097/MD.0000000000045803

**Published:** 2025-11-21

**Authors:** Mengying Wang, Wenqing Ruan, He Li, Wei Yuan, Jiangang Song, Jian Wang

**Affiliations:** a Department of Anesthesiology, Shuguang Hospital Affiliated to Shanghai University of Traditional Chinese Medicine, Shanghai, China.

**Keywords:** beginners, endotracheal intubation, simulated manikin, video laryngoscope

## Abstract

The emergence of videolaryngoscopy has transformed airway management, being widely adopted for tracheal intubation, and enhancing glottic visualization, especially in complex scenarios. Continuous research is crucial to evaluate the efficacy of various designs. This study aims at evaluating the usability and safety of videolaryngoscopes in novice intubation simulations. This study compares the impact of LANJA, UE, and VDO video laryngoscopes on simulated endotracheal intubation by beginners. This non-randomized concurrent controlled simulation study involved 29 medical student volunteers with no prior endotracheal intubation experience. Following theoretical training by experienced anesthesiologists, participants watched intubation demonstration videos and engaged in hands-on practice. Using LANJA, UE, and VDO video laryngoscopes in random order, participants performed endotracheal intubation on a simulated airway mannequin. Observers recorded intubation procedure videos, analyzing intubation time, success rate, and laryngoscope exposure grading. Significant differences were observed in the time required for glottic exposure with the 3 laryngoscopes (intubation visualization time; LANJA: 5.47 ± 2.84; UE: 9.29 ± 2.88; VDO: 8.97 ± 2.99, *P* = .001). The endotracheal tube placement time (visualization and fixation time) did not differ significantly among the laryngoscopes (LANJA: 25.21 ± 7.35; UE: 25.04 ± 7.75; VDO: 27.66 ± 3.80, *P* = .31). Overall intubation success rates varied significantly among the 3 laryngoscopes (LANJA: 89.5%, UE: 78.9%, VDO: 63.2%, *P* = .002); within Cormack–Lehane grades, LANJA showed the highest percentage in Grade I (68.8%). LANJA video laryngoscope demonstrated a higher success rate in guiding endotracheal intubation, reducing glottic exposure time, and is suitable for use by beginners.

## 1. Introduction

With the widespread adoption of video laryngoscopes, the safety, accuracy, and success rates of endotracheal intubation have significantly improved.^[1]^ Currently, various video laryngoscopes are employed in clinical practice, categorized into 3 types based on structural features and intubation forms: Macintosh-style video laryngoscopes; hyperangulated video laryngoscopes, and video laryngoscopes with an integrated endotracheal tube guidance channel.^[2]^ Different laryngoscope structures may have an impact on intubation time, patient cardiovascular responses, or intubation complications.^[3,4]^

Macintosh-style video laryngoscopes and hyperangulated video laryngoscopes are 2 commonly used endotracheal intubation devices, each with distinct design and usage characteristics. The usage of Macintosh-style video laryngoscopes for endotracheal intubation is somewhat similar to using direct Macintosh laryngoscopes. During intubation, the tongue and soft tissues need to be displaced upward to visualize the glottis, and pre-shaping of the endotracheal tube is not necessary, making tube insertion relatively easier.^[5]^ In contrast, hyperangulated laryngoscopes have a greater curvature, allowing clear visualization of the glottis without significant changes to the position of the tongue and soft tissues. This is advantageous in situations with limited mouth opening, restricted neck movement, or limited head extension. However, obtaining a good glottic view with high-angle video laryngoscopes may increase the difficulty of endotracheal tube insertion,^[6]^to make the intubation process easier, the usage of a stylet to shape the tube is commonly required.

This study reports a novel video laryngoscope, the LANJA laryngoscope (Patent Number: CN208988826U). In current clinical practice, traditional high-angle video laryngoscopes often encounter challenges during tracheal intubation, such as limited visibility due to the angle of the device, which can lead to difficulties in glottic exposure and increased time for successful intubation, especially in patients with challenging airway anatomies.The LANJA laryngoscope is specifically designed to address these issues. Its distal end is angled at approximately 90°, and its “L”-shaped design conforms to the anatomical structure of the oropharynx in a neutral position. By optimizing the design of the camera and the probe, this laryngoscope significantly enhances the exposure of the glottis. It provides a broader operational space and a clearer visual field for intubation procedures, thereby potentially reducing intubation time and improving the success rate, even in difficult airway scenarios.

Based on the structural improvement of this hyperangulated video laryngoscope, we hypothesize that the application of the novel hyperangulated video laryngoscope is superior to that of the Macintosh-style video laryngoscope, especially for normal airways.

## 2. Materials and methods

The study included volunteer residents and medical students with no prior experience in endotracheal intubation. The training was conducted over a period of 7 days, with each training session lasting approximately 4 hours per day. This duration was carefully chosen to allow participants sufficient time to absorb the theoretical knowledge, practice the intubation techniques, and become familiar with the equipment and procedures involved. It began with a detailed theoretical overview, which included explanations of the airway anatomy, the indications and contraindications for tracheal intubation, and the principles of video-laryngoscopy. Participants were also taught about the different types of video laryngoscopes, their features, and how they differ from traditional direct laryngoscopes. Next, the focus shifted to intubation techniques. Experienced anesthesiologists, each with at least 10 years of clinical practice in airway management, demonstrated the proper use of the LANJA, UE, and VDO video laryngoscopes. All participants were fully informed prior to recruitment. Since no patients have participated in this study, informed consent and ethical approval are not required.

**Figure 1.**
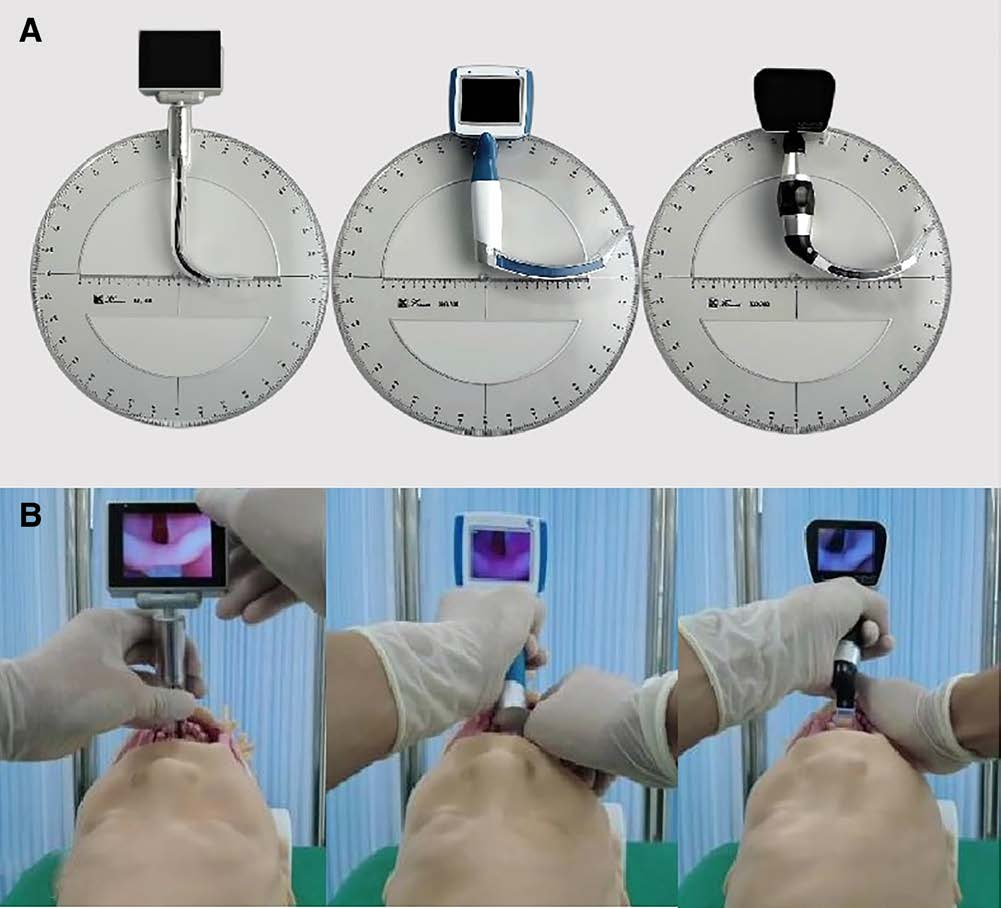
Comparison of 3 Laryngoscopes. (A) Comparison of the structures of LANJA laryngoscope (left), UE laryngoscope (middle), and VDO laryngoscope (right). (B) Comparison of glottic exposure with the 3 laryngoscopes.

Volunteers were randomly assigned to use 3 different laryngoscopes in simulated endotracheal intubation, including one hyperangulated laryngoscope (UE-TDC3, Zhejiang UeMedical Equipment Co., Ltd., Xianju, China), another hyperangulated laryngoscope (VDO-100A, Shanghai Jingren Medical Technology Co., Ltd., Shanghai, China), and the LANJA laryngoscope (LJ-N700, Shanghai Lanjia Medical Technology Co., Ltd., Shanghai, China). Comparison of 3 types of video laryngoscopes is demonstrated in Figure 1. To minimize learning bias, random sequences of laryngoscope use were generated using the http://www.random.org/ website tool, and the laryngoscope usage order was randomly assigned a numerical identifier. Volunteers conducted normal endotracheal intubation on a simulated airway manikin (Model: PLHX1002, Pulin Medical Technology Co., Ltd., Tangshan, China) using 3 laryngoscopes in random order. This manikin is designed to closely mimic the human airway. It has a life-sized head and neck, with a realistic oral cavity, pharynx, larynx, and trachea. The soft-tissue parts like the tongue are flexible, offering a tactile experience akin to real patients. It has adjustable difficulty settings. The tongue position can be altered to be more anterior or posterior. An anterior tongue can block the view of the larynx, increasing intubation challenge. The pharyngeal soft-tissue compliance is also adjustable; less compliant tissues simulate conditions like pharyngeal edema, adding resistance during laryngoscope insertion. Additionally, it can present different Cormack–Lehane grades of laryngeal view, enabling trainees to practice in scenarios with varying glottic visibility. A 7.5mm endotracheal tube and a hand-controlled noninvasive ventilation device were used, and tube position was confirmed by an assistant with recorded procedure videos and documented data.

The primary outcomes of this study included intubation time (in seconds), divided into 3 phases: intubation visualization time (IVT) – the time from the laryngoscope tip passing the incisors to the exposure of the vocal cords which is able to perform Cormack–Lehane (C–L) grading; visualization and fixation time (VFT) – the time from vocal cord exposure to successful intubation and withdrawal of the laryngoscope; fixation and ventilation time (FVT) – the time from successful intubation to the first ventilation. Secondary outcomes encompassed intubation success rate (%) and laryngeal exposure grading (C–L grading), which includes 4 levels: Grade 1, fully exposing the vocal cords, visible anterior and posterior commissures; Grade 2, visualization of the epiglottis and posterior part of the vocal cords; Grade 3, only the epiglottis or the anterior part is visible; and Grade 4, unable to see the epiglottis and vocal cords, only the soft palate is visible. We defined intubation failure as esophageal intubation or a total intubation time (IVT + VFT) exceeding 90 seconds.A successful intubation was defined by meeting the following criteria. First, the endotracheal tube must be correctly placed in the trachea, confirmed by chest rise observation, bilateral lung auscultation, and (if available) detection of exhaled carbon dioxide. Second, the total intubation time (IVT + VFT) should not exceed 90 seconds. Third, there should be no esophageal intubation. Only when all 3 conditions are met is it considered a successful intubation.

An independent observer, blinded to the study, analyzed operation videos to evaluate intubation time, success rate, and laryngeal exposure grading.

In a pilot study, the time from intubation completion to the first ventilation using LANJA and UE laryngoscopes was 18.27 ± 10.79 (S) and 20.72 ± 9.82 (S), respectively. To detect a 33% difference in intubation time, with a statistical power of 0.95 and assuming a 20% dropout rate, a sample size of 28 volunteers was estimated.

Statistical analysis was performed using SPSS 26.0 (SPSS Inc., Chicago). Kruskal–Wallis test was used for continuous variables to compare different laryngoscope types in each time interval. Chi-square test was employed for comparing categorical variables (such as intubation success rate), with *P* < .05 considered statistically significant. Post hoc analysis for Mann–Whitney test was conducted using Bonferroni correction, and *P* < .017 was considered significant.

## 3. Results

The participants in this study consisted of medical students and resident physicians, all of whom had no prior experience in endotracheal intubation. In March 2022, a total of 29 participants were recruited, comprising 16 males (55.2%) and 13 females (44.8%), with ages ranging from 23 to 30 years. Demographic information of the volunteers is presented in Table 1.

**Table 1:** Demographic information.

	Male	Female
Number, n (%)	16 (55.2)	13 (44.8)
Age (mean ± SD)	26 ± 2.5	25 ± 3.2
Experience in endotracheal intubation	No	No

SD = standard deviation.

In the overall comparison of the 3 laryngoscopes, significant differences were observed in the IVT time (LANJA: 5.47 ± 2.84; UE: 9.29 ± 2.88; VDO: 8.97 ± 2.99, *P* = .001) and the FVT time (LANJA: 5.61 ± 1.88; UE: 6.95 ± 2.82; VDO: 4.95 ± 1.80, *P* = .004). However, there was no significant difference in the VFT time among the 3 laryngoscopes (LANJA: 25.21 ± 7.35; UE: 25.04 ± 7.75; VDO: 27.66 ± 3.80, *P* = .31).

In the overall comparison of intubation success rates among the 3 laryngoscopes, significant differences were noted (LANJA: 89.5%, UE: 78.9%, VDO: 63.2%, *P* = .002). Among the 3 laryngoscopes, LANJA had the highest proportion of C–L Grade I of 68.8% (Table 2).

**Table 2:** Comparison of Cormack–Lehane (CL) grading for endotracheal intubation with 3 laryngoscopes.

Device	C–L Grade, n (%)			
	I	II	III	IV
LANJA	22 (68.8)	8 (25)	2 (6.3)	0 (0)
UE	18 (56.3)	9 (28.1)	4 (12.5)	1 (3.1)
VDO	15 (46.9)	11 (34.4)	5 (15.6)	1 (3.1)
Post hoc analysis	*P* value*			
LANJA vs UE	.001	1	.054	.076
LANJA vs VDO	.001	.551	.834	0
UE vs VDO	1	.495	.005	.01

C–L grade = Cormack–Lehane grading, intubation time.

* Post hoc analyses were conducted with Bonferroni correction, and *P* < .017 was considered significant.

In pairwise comparisons, the IVT time with LANJA laryngoscope was significantly shorter than with UE and VDO laryngoscopes (*P* = .001). And there was no significant difference in IVT time between UE and VDO laryngoscopes (*P* = 1.000). The VFT time showed no significant difference among the 3 laryngoscopes (LANJA vs UE, *P* = 1.000; LANJA vs VDO, *P* = .551; UE vs VDO, *P* = .495). In the FVT time comparison, there was no significant difference between LANJA laryngoscope and UE or VDO laryngoscopes (LANJA vs UE, *P* = .054; LANJA vs VDO, *P* = .834). However, VDO laryngoscope had a significantly shorter FVT time than UE laryngoscope (UE vs VDO, *P* = .005).

In pairwise comparisons, both LANJA and UE laryngoscopes had higher intubation success rates than VDO laryngoscope, with significant differences (LANJA vs VDO, *P* = .0001; UE vs VDO, *P* = .01). There was no significant difference in intubation success rates between LANJA and UE laryngoscopes (LANJA vs UE, *P* = .076), as shown in Table 3.

**Table 3:** Comparison of the success rates of endotracheal intubation data with 3 laryngoscopes.

Device	IVT	VFT	FVT	Success rate %
LANJA	5.47 ± 2.84	25.21 ± 7.35	5.61 ± 1.88	89.5
UE	9.29 ± 2.88	25.04 ± 7.75	6.95 ± 2.82	78.9
VDO	8.97 ± 2.99	27.66 ± 3.80	4.95 ± 1.80	63.2
*P* value	.001	.31	.004	.002

Continuous variables are presented as mean ± standard deviation. *P*-values were calculated through ANOVA, and *P* < .05 was considered significant. Three phases: IVT – time from laryngoscope tip passing incisors to exposure of vocal cords and ability to perform CL grading; VFT – time from vocal cord exposure to successful intubation and withdrawal of laryngoscope; and FVT – time from successful intubation to first ventilation.

FVT = fixation and ventilation time, IVT = intubation visualization time, VFT = visualization and fixation time.

## 4. Discussion

Tracheal intubation duration serves as a crucial metric for evaluating the difficulty of laryngoscope devices and tracheal intubation procedures. Discrepancies in the definition of tracheal intubation duration have been noted in studies comparing various video laryngoscopes.^[7]^ In this investigation, the time elapsed from the insertion of the laryngoscope into the oral cavity until confirmation of the endotracheal tube’s proper placement is defined as the intubation duration, and we further segmented the time duration into 3 phases^[8]^: IVT: the duration from the laryngoscope tip passing the incisors to the exposure of the vocal cords and the ability to perform C–L grading; VFT: the duration from vocal cord exposure to successful tube insertion and subsequent withdrawal of the laryngoscope; and FVT: the duration from successful tube insertion to the initiation of the first ventilation. These phases reflect the time required using different laryngoscopes during critical processes such as vocal cord exposure and endotracheal tube placement. Vocal cord exposure and tube insertion are 2 pivotal steps in tracheal intubation using a laryngoscope. The degree of vocal cord exposure and the smoothness of tube insertion are closely correlated with intubation duration and success rates.^[6]^ This study revealed significant differences among novice users in IVT and FVT when employing 3 different laryngoscopes (*P*-values of .001 and .004, respectively), while no significant difference was observed in VFT (*P*-value = 0.31). The LANJA laryngoscope exhibited the shortest time required for vocal cord exposure. In this experiment, most novices using the LANJA laryngoscope achieved vocal cord exposure within 10 to 20 seconds, whereas the use of UE and VDO laryngoscopes took 15 to 30 seconds, aligning closely with previously reported times for Macintosh video laryngoscope and hyperangulated video laryngoscope.^[9]^ However, Niforopoulou et al study^[10]^ suggested that despite excellent visualization of vocal cord structures, intubation using a video laryngoscope may still encounter difficulties. This might be attributed to the indirect display of the target through the camera and variations in the angles of different laryngoscope blades.

In recent years, with the widespread adoption of video laryngoscopes, there has been a gradual diversification in the curvature of laryngoscope blades. This evolution ranges from the standard Macintosh laryngoscope blade with an approximately 30° curvature to intermediate angle blades with 30° to 60° curvature and hyperangulated blades with a curvature >60°. The varied thickness and shapes of these blades contribute to performance disparities.^[1,11,12]^ The UE and VDO video laryngoscopes fall into the category of intermediate angle blades, while the LANJA video laryngoscope features a hyperangulated blade structure, with blade shapes and angles more closely resembling traditional Macintosh laryngoscope blades. Studies indicate that anesthesiologists with experience using laryngoscopes typically favor the use of direct Macintosh laryngoscopes during their learning and training phases. Proficiency with direct laryngoscopes does not necessarily translate to proficiency with video laryngoscopes, and experience with Macintosh laryngoscopes does not necessarily expedite the learning curve for video laryngoscopes.^[13,14]^ In this study, participants had no prior experience with any type of laryngoscope. All 3 laryngoscopes were used by the same group of participants, and compared to the LANJA laryngoscope, the intubation times for UE and VDO laryngoscopes were longer, potentially due to differences in laryngoscope structure.

This study revealed that the LANJA video laryngoscope exhibited the highest intubation success rate among novice users (89.5%), while the success rates for UE and VDO laryngoscopes were 78.9% and 63.2%, respectively. There was a significant difference in success rates compared to the other 2 video laryngoscopes (*P* = .002). Currently, there is a lack of robust research comparing the intubation success rates of different video laryngoscopes. Video laryngoscopes, when compared to direct laryngoscopes, have been shown to enhance the success rate of tracheal intubation, especially for beginners. A comprehensive review and meta-analysis of 8 studies found that in prehospital emergency intubation procedures, the use of video laryngoscopes significantly improved overall success rates compared to direct laryngoscopes.^[15]^ Further data exhibit that video laryngoscopes derived from Macintosh laryngoscopes have success rates ranging from 69% to 100% in both normal and difficult airways.^[7,15,16]^ Regarding C–L grading for laryngeal exposure, using the LANJA laryngoscope for intubation resulted in the highest proportion of Grade I exposure, at 68.8%. Further research is needed to clarify the correlation between different laryngoscope structures and C–L grading.

The use of standardized simulated patients for video laryngoscope comparative studies has been proven to be a reliable alternative to clinical trials.^[17]^ This approach can reveal the strengths and weaknesses of video laryngoscopes with different structures during normal intubation processes, providing more evidence for clinical intubation technique selection and training.

### 4.1. Limitations

However, this study has some limitations. Firstly, simulated patients only mimic normal airway structures and cannot replicate real physiological conditions, such as body temperature, secretions, bleeding, and mist, which may increase the challenges of airway management.^[18]^ Secondly, this study only involved simulations of normal airway intubation and did not simulate common difficult airway scenarios encountered in clinical practice. Therefore, the study conclusions cannot be generalized to beginners dealing with difficult airway intubation. Thirdly, human model studies cannot fully assess participants’ mental stress, cognitive deficiencies, and other factors. In actual clinical practice, the use of video laryngoscopes requires the operator’s hand-eye coordination, understanding of image anatomy, familiarity with different devices, and their specific operating techniques. Further research should consider subjective feedback and evaluations from operators to more comprehensively assess the operational experience and effectiveness of video laryngoscopes. Similar studies targeting difficult airways to evaluate the effects of different laryngoscopes in managing difficult airways and reducing complications would also be clinically meaningful.

## 5. Conclusions

In summary, the LANJA video laryngoscope demonstrates a higher success rate, shorter vocal cord exposure time, and improved vocal cord exposure effectiveness among novices performing simulated patient tracheal intubation. The LANJA video laryngoscope holds significant clinical application value.

## Author contributions

**Conceptualization:** Jian Wang.

**Data curation:** Mengying Wang, Wenqing Ruan, He Li, Wei Yuan, Jiangang Song.

**Formal analysis:** Mengying Wang, He Li, Wei Yuan, Jiangang Song.

**Investigation:** Wenqing Ruan, Wei Yuan.

**Project administration:** Jian Wang.

**Software:** He Li, Wei Yuan.

**Resources:** Jian Wang.

**Writing – original draft:** Mengying Wang, Wenqing Ruan, Wei Yuan, Jiangang Song.

**Writing – review & editing:** Mengying Wang, Wenqing Ruan, He Li, Wei Yuan, Jiangang Song, Jian Wang.
